# Mode of action of *Akkermansia muciniphila* in the intestinal dialogue: role of extracellular proteins, metabolites and cell envelope components

**DOI:** 10.20517/mrr.2023.05

**Published:** 2023-03-13

**Authors:** Anneleen Segers, Willem M. de Vos

**Affiliations:** ^1^Laboratory of Microbiology, Wageningen University & Research, Wageningen 6708 WE, The Netherlands.; ^2^Human Microbiome Research Program, Faculty of Medicine, University of Helsinki, Helsinki 00014, Finland.

**Keywords:** *Akkermansia muciniphila*, Amuc_1100, pilus-associated signaling protein, P9, extracellular and secreted proteins, metabolites, cell envelope components

## Abstract

*Akkermansia muciniphila* is a promising next-generation beneficial microbe due to its natural presence in the mucus layer of the gut, its symbiotic ability to degrade mucus, and its capacity to improve the intestinal barrier function. *A. muciniphila* is able to counteract weight gain and immuno-metabolic disturbances in several animal models. Many of these disorders, including obesity and auto-immune diseases, have been associated with decreased gut barrier function and consequent increased inflammation. Since *A. muciniphila* was found to normalize these changes and strengthen the gut barrier function, it is hypothesized that other beneficial effects of *A. muciniphila* might be caused by this restoration. In search for *A. muciniphila’s* mode of action in enhancing the gut barrier function and promoting health, we reasoned that secreted components or cell envelope components of *A. muciniphila *are interesting candidates as they can potentially reach and interact with the epithelial barrier. In this review, we focus on the potential mechanisms through which *A. muciniphila *can exert its beneficial effects on the host by the production of extracellular and secreted proteins, metabolites and cell envelope components. These products have been studied in isolation for their structure, signaling capacity, and in some cases, also for their effects in preclinical models. This includes the protein known as Amuc_1100, which we here rename as pilus-associated signaling (PAS) protein , the P9 protein encoded by Amuc_1631, the short-chain fatty acids acetate and propionate, and cell envelope components, such as phosphatidylethanolamine and peptidoglycan.

## INTRODUCTION

**Figure 1:**
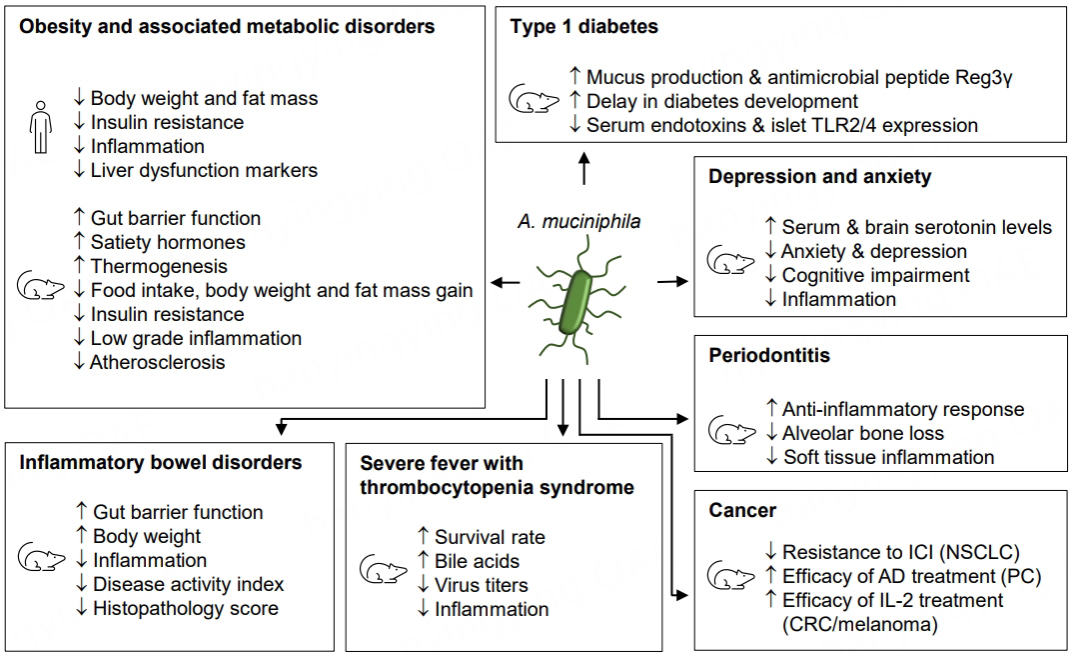
Main effects of treatment with *A. muciniphila* in animal models of different diseases. See Table 1 for an overview of the -mainly preclinical- studies. AD: Androgen deprivation; CRC; colorectal cancer; ICI: immune checkpoint inhibitors; NSCLC: non-small cell lung cancer; PC: prostate cancer; TLR: Toll-like receptor.

*A. muciniphila* is an anaerobic Gram-negative bacterium that is abundantly present in the gastrointestinal tract of humans and several animals^[1,2]^. *A. muciniphila *is a promising next-generation beneficial microbe due to its natural presence in the mucus layer of the gut, its symbiotic ability to degrade mucus, and its capacity to improve the intestinal barrier function, which is fundamental to many diseases^[3,4]^. Ever-increasing amounts of studies have shown a negative correlation between fecal relative levels of *A. muciniphila *and diseases, such as inflammatory bowel diseases (IBD), obesity and type 2 diabetes and its related cardiometabolic disorders, and other inflammatory or auto-immune diseases, such as asthma and type 1 diabetes^[5-13]^. In recent years, *A. muciniphila *has been shown to counteract weight gain, gut barrier function disruptions and metabolic disturbances in several animal models of these diseases^[10,14-17] ^[Figure 1 and Table 1]. However, not all models respond similarly and a recent study suggests that there may be sex differences that need to be further addressed^[18]^.

**Table 1:** Effect of treatment with *A. muciniphila* cells, proteins or metabolites in animal models of different diseases

**Disease modeled**	**Animal model**	**Treatment**	**Effect**	**Reference**
Obesity and related cardiometabolic disorders	Multiple	10^8 ^- 5 × 10^9^ CFU of live or pasteurized *A. muciniphila* (4 weeks - 10 months)	↓ Food intake, body weight and fat mass gain↓ Insulin resistance↓ Low-grade inflammation↑ Gut barrier function	[106,107]
	HFD-fed C57BL/6J mice	Daily oral gavage (5 weeks) with 3 μg PAS*	↓ Body weight gain↓ Plasma high-density lipoproteins↑ Glucose tolerance↑ Gut barrier function	[14]
	HFD-fed SD rats treated with streptozotocin	Daily oral gavage (8 weeks) with 3 μg PAS*	↓ Body weight gain↑ Gut barrier function	[55,56]
	HFD-fed zebrafish	Administration in feed (4 weeks) oflive *Lactococcus lactis ZHY1* with surface-expressed PAS	↓ Body weight gain↓ Hepatic steatosis↑ Gut barrier function	[57]
	HFD-fed C57BL/6J mice	Daily oral gavage (8 weeks) with 100 μg P9*	↓ Body weight gain, fat mass gain↑ Thermogenesis, *Gcg* expression↑ Glucose tolerance	[81]
	HFD-fed female C57BL/6J mice	Fecal microbiota transplantation from estradiol-treated lean female mice, supplemented with 2 × 10^8^ live *A. muciniphila*	*A. muciniphila* treatment had no or even negative effects:= Food intake, energy expenditure↑ Fasting glucose levels	[18]
Inflammatory bowel disorders	C57BL/6J treated with DSS	Daily oral gavage (2 weeks) with 1.5 × 10^8[59]^ or 3 × 10^9[108]^ CFU of live *A. muciniphila*Daily oral gavage (8 weeks) with 2 × 10^8^ CFU of live *A. muciniphila*^[16]^Daily oral gavage (1 week) with 5 × 10^8^ CFU of live *A. muciniphila* or 100 mg EV^[47]^	↓ Body weight loss↓ Histopathology score↑ Gut barrier function↓ Inflammation	[16,47,59,108]
	C57BL/6J treated with DSS	Daily oral gavage (2 weeks) with 3 μg PAS*	↓ Disease activity index↓ Histopathology score↓ Inflammation	[59]
	C57BL/6J treated with DSS and azoxymethane (CAC model)	Daily oral gavage (2 weeks) with 3 μg PAS* or 1.5 × 10^8 ^CFU CFU of live *A. muciniphila*	↓ Disease activity index↓ DNA damage, cell apoptosis, abnormal proliferation↑ Delay in tumor formation	[59]
	C57BL/6J treated with DSS	Daily oral gavage (12 days) with 3.6 μM Amuc_1409*	↑ Body weight↓ Histopathology score↑ Intestinal stem cell markers	[85]
	C57BL/6J treated with DSS	Daily oral gavage (3 weeks) of 20 μg Amuc_2109*	↓ Body weight loss↓ Disease activity index↑ Gut barrier function↓ Inflammation	[86]
	C57BL/6J treated with 5-Fluorouracil (mucositis model)	Daily oral gavage (2 weeks) with 100 μgPAS* or 1.5 × 10^9^ CFU of live *A. muciniphila*	↓ Diarrheal toxicity↓ Histopathology score↑ Gut barrier function↓ Inflammation	[72]
Type 1 Diabetes	NOD mice	Daily oral gavage (7 weeks) with 2 × 10^8^ CFU of live *A. muciniphila*	↑ Mucus production and antimicrobial peptide Reg3γ↓ Serum endotoxins, TLR2/4 expression on islets↑ Delay in diabetes development	[15]
Periodontitis	C57BL/6J treated with *Porphyromonas gingivalis*	Subcutaneous injection with 10^9^ CFU of live *A. muciniphila*	↓ Alveolar bone loss↓ Soft tissue inflammation↑ Anti-inflammatory response	[75,76]
	C57BL/6J treated with *Porphyromonas gingivalis*	3 x per week oral gavage (6 weeks) with 6 μg PAS* or 10^9^ CFU of live *A. muciniphila*	↓ Alveolar bone loss↓ M1/M2 macrophage ratio	[76]
Severe fever with thrombocytopenia syndrome	C57BL/6J mice treated with Ab, anti-IFNAR1 IgG and SFTSV	Daily oral gavage (1 week) with *A. muciniphila* or *A. muciniphila* metabolite harmaline	↑ Survival rate↓ Titers of SFTSV↓ Inflammation↑ Bile acids	[92,93]
Depression and anxiety	C57BL/6J mice under CUMS	Daily oral gavage (6 weeks) with 80 μg PAS^#^	↓ Depression-like behavior↑ Serotonin levels in brainstem↓ Cognitive impairment	[69]
	C57BL/6J mice treated with Ab	Daily oral gavage (2 weeks) with 100 μg PAS^#^ or 1.5 × 10^9^ CFU of live *A. muciniphila*^[70]^Daily oral gavage (3 weeks) with 80 μg PAS^#^ or 80 μg PAS^Δ80[71]^	↓ Anxiety and depression-like behavior↓ Cognitive impairment↑ Serotonin levels in serum and hippocampus↓ Inflammation	[70,71]
Neurodegenerative diseases	G93A SOD1-Tg mice (ALS model)	Oral gavage of *A. muciniphila* or systemic administration via osmotic pumps of *A. muciniphila* metabolite nicotinamide	↑ Motor function	[23]
Cancer	Different murine non-small cell lung cancer models with fecal microbiota transplanted from patients without *A. muciniphila*	5 x oral gavage of 10^8^ CFU of live *A. muciniphila*: 24 h before the first injection of ICI and four times on the same day of ICI	↓ Dysbiosis associated with ICI resistance↓ ICI resistance	[30]
	Murine colorectal cancer model(Balb/c mice) treated with IL-2	Oral gavage of 10^8^ CFU of live or pasteurized *A. muciniphila* or 10 μg PAS* every 3 days for 25 days	↑ Efficacy of IL-2 immunotherapy treatment↑ Gut barrier function	[32]
	Murine melanoma model (Balb/c mice) treated with IL-2	Oral gavage of 10^8^ CFU of live or pasteurized *A. muciniphila* or 10 μg PAS* every 3 days for 23 days	↑ Efficacy of IL-2 immunotherapy treatment↑ Gut barrier function	[32]
	Murine prostate cancer model (FVB/NJ mice)	2 x oral gavage of 10^8^ CFU of pasteurized *A. muciniphila*: 24 h before androgen deprivation therapy and 6 h after	↑ Efficacy of androgen deficiency treatment	[34]
	Several murine colorectal cancer models	150 μg/kg Amuc_2172* 2 x per week via:- Intraperitoneal injection (14 weeks)- Coated nanoparticles, intravenously injected (24 days)	↑ HSP70 transcription and secretion↑ CTL immune activity↓ Tumorgenesis	[88]

Ab: Antibiotics; ALS: amyotrophic lateral sclerosis; CAC: colitis-associated colorectal cancer; CFU: colony forming units; CUMS: chronic unpredictable mild stress; DSS: dextran sulfate sodium; EV: extracellular vesicles; HFD: high-fat diet; NOD: non-obese diabetic; SD rats: Sprague Dawley rats; SFTSV : Severe fever with thrombocytopenia syndrome virus; TLR: Toll-like receptor.

In contrast to IBD, obese and cardiometabolic patients, where *A. muciniphila *is depleted, several studies showed that *A. muciniphila* is more abundant in the microbiota of patients with neurological disorders or stroke^[19-21]^. However, as discussed recently, the latter suffer from many confounders, including medication and transit time reduction^[3]^. Moreover, there is no evidence that supports a direct role of *A. muciniphila *in promoting progression or symptoms of these diseases. In contrast, animal and human intervention studies support that the increased abundance of *A. muciniphila* leads to a protective response to promote recovery in multiple sclerosis (MS) and amyotrophic lateral sclerosis (ALS)^[3,22,23]^*. *Metformin, a drug widely used for treatment of diabetes, was shown to have anti-inflammatory effects and might aid in the treatment of MS^[24,25]^. Metformin use increases the relative abundance of *A. muciniphila*^[26]^, further supporting a potential protective or curative role of *A. muciniphila.*

*A. muciniphila *might be able to aid in the treatment of several cancers. *A. muciniphila *was found to be increased in patients that were responding to immune checkpoint inhibitor (ICI) treatment for non-small cell lung cancer (NSCLC), hepatocellular carcinoma or renal cell carcinoma, while patients with progressive disease showed low relative *A. muciniphila *abundance^[27-29]^. However, a decrease in overall survival rate was found in patients with an overabundance of *A. muciniphila*, indicating that “normal” levels of *A. muciniphila* might be a biomarker for optimal potential response to ICI. In a mouse model of non-responding NSCLC patients, where fecal material of patients without detectable *A. muciniphila* was transferred, treatment with *A. muciniphila* strain *Akkp2261 *could restore ICI response^[30]^. In advanced cutaneous melanoma patients, *A. muciniphila *levels were also generally higher in ICI responders, but meta-analysis showed that this was not consistent in different studies, highlighting the complexity of the role of gut microbiota in ICI response^[31]^. Nevertheless, in IL-2 immunotherapy, co-treatment with *A. muciniphila *strengthened IL-2 antitumor effects in both subcutaneous melanoma and colorectal tumor-bearing mice^[32]^. In prostate cancer patients, *A. muciniphila *showed a positive correlation with response to androgen deprivation therapy^[33,34]^. In addition, in prostate cancer-bearing mice, oral gavage with *Akkp2261 *improved the efficacy of androgen deprivation therapy^[34]^.

**Figure 2:**
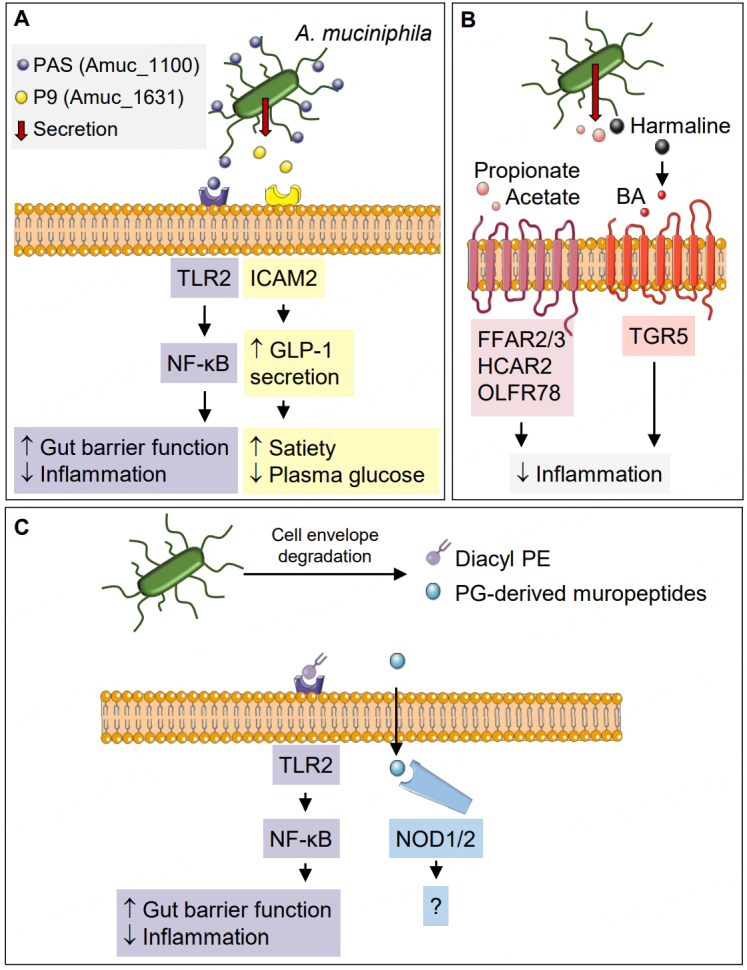
*A. muciniphila* and its (A) secreted and outer membrane proteins, (B) metabolites and (C) cell envelope fragments with their associated receptors and putative effects on the host. Diacyl PE: Diacyl phosphatidylethanolamine; FFAR2/3: free fatty acid receptor 2/3; HCAR2: hydroxycarboxylic acid receptor 2; ICAM2: intercellular adhesion molecule 2; NOD1/2: nucleotide-binding oligomerization domain 1/2; NF-κB: nuclear factor kappa-light-chain-enhancer of activated B cells; OLFR78: olfactory receptor 78; PG: peptidoglycan; TGR5: Takeda G protein-coupled receptor 5; TLR2: Toll-like receptor 2; PAS: pilus-associated signaling protein. This figure was partly generated using Servier Medical Art, licensed under a Creative Commons Attribution 3.0 unported license.

Many of these disorders have been associated with decreased gut barrier function and consequent increased inflammation, which in turn can cause symptoms ranging from weight gain and hepatic steatosis to cognitive disorders^[35]^. Recently it has been hypothesized that a defective barrier function is also the base for auto-immune diseases and other chronic conditions^[36]^. Increased gut permeability can be caused by increased paracellular epithelial permeability due to decreased tight and adherens junctions, decreased mucus thickness, changes in gut microbiota and/or dysfunctional gut-associated immune responses to pathogens. Since *A. muciniphila *was found to counteract these changes and strengthen the gut barrier function, it is tempting to hypothesize that other beneficial effects of *A. muciniphila *might be caused by this restoration. In search for *A. muciniphila’*s mode of action in enhancing the gut barrier function and promoting health, secreted components or outer membrane components of *A. muciniphila *are likely candidates as they can potentially reach and interact with the epithelial barrier. In this review, we will therefore focus on the potential mechanisms through which *A. muciniphila *can exert its beneficial effects on the host by the production of extracellular and secreted proteins, metabolites and cell envelope components, which have been studied in isolation for their structure, signaling capacity, and in some cases, also for their effects in preclinical models [Figure 2]. This includes the protein known as Amuc_1100, which we here rename as pilus-associated signaling (PAS) protein, as will be discussed below, the P9 protein encoded by the gene with locus tag Amuc_1631, the short-chain fatty acids (SCFAs) acetate and propionate, and cell envelope components, such as phosphatidylethanolamine (Diacyl PE), ornithine lipids and peptidoglycan (PG). All these mode of action studies have been performed with the *A. muciniphila* type strain Muc^T^^[37]^. This strain has been used in most preclinical and human intervention studies, as reviewed recently^[3]^. It has also been extensively characterized at the genome, proteome and transcriptome levels^[38,39]^. This body of knowledge facilitates further comparative analysis of its functions, as will be discussed below.

Since its isolation in 2004, the number of publications related to *A. muciniphila *has been rapidly increasing^[40]^. Consequently, most insight derives from recent studies and a complete picture is only emerging. In 2016, the seminal study by Plovier *et al. *provided a first major step in elucidating the mode of action of *A. muciniphila*^[14]^*.* While autoclaving *A. muciniphila* abolishes its beneficial effects^[10]^, it was found that less extreme pasteurization at 70 °C for 30 minutes, which inactivates life in this Gram-negative bacterium but limits the denaturation of some proteins, did not abolish its beneficial effects. High-fat diet (HFD) fed mice that were treated with pasteurized *A. muciniphila *showed significantly lower fat mass and decreased weight gain, glucose intolerance and insulin concentration compared to non-treated HFD mice. In addition, plasma levels of the satiety hormone leptin and the mean adipocyte diameter were decreased by treatment with pasteurized *A. muciniphila*, while treatment with live *A. muciniphila* did not affect leptin levels and adipocyte size. The reduction in body weight gain in pasteurized *A. muciniphila*-treated mice might partially be explained by lower energy absorption, since a higher fecal caloric content was observed in this group. Taken together, this data indicates that pasteurization enhances the beneficial effects of *A. muciniphila *on HFD-induced symptoms. This suggests that accessibility of specific beneficial bacterial proteins, such as Amuc_1100 (see below) which is present on the outer membrane, or other compounds, is increased by pasteurization^[14]^.

Importantly, similar results were obtained in a first human intervention study with overweight and diabetic patients. Administration of live as well as pasteurized *A. muciniphila *improved insulin sensitivity, reduced insulinemia and plasma total cholesterol and decreased body weight and fat mass^[41]^. Moreover, pasteurized *A. muciniphila *was found to be well-tolerated in these proof-of-concept studies^[14,41]^. In addition, following extensive toxicological analysis, the pasteurized *A. muciniphila *cells were found to be safe for human consumption. This resulted in passing the novel food regulations of the European Food Safety Authority, which is known to have one of the world’s strictest safety regimens^[42]^.

## EXTRACELLULAR VESICLES

Since pasteurized *A. muciniphila *seems to retain its beneficial effects on metabolic disorders, it seems counterintuitive that extracellular vesicles (EV) secreted by *A. muciniphila *would have similar beneficial effects on their host. Nevertheless, EV, also named outer membrane vesicles, of pathogenic bacteria are important for communication with the host. It is unclear whether EV of *A. muciniphila* have the same function.

EV of *A. muciniphila *were shown to contain proteins covering a wide range of molecular weights, from 11 to 245 kDa^[43]^. In HFD-fed mice, they improved the gut barrier function, reduced body weight gain and improved glucose tolerance. The effect of EV was claimed to be more pronounced than the effect of live or pasteurized *A. muciniphila *cells, although a careful cell- and mass-based comparison is lacking^[44-46]^. In addition, DSS-fed mice treated with EV of *A. muciniphila *showed similar health improvements compared to mice treated with *A. muciniphila. *Weight loss and the disease activity index were reduced, colon length was increased, and epithelial stability and inflammatory cell infiltration of the colon wall was ameliorated^[47]^. *A. muciniphila* and its EV could also play a role in the prevention of liver fibrosis. Both live and pasteurized *A. muciniphila*, as well as its EV, could prevent HFD/CCL4-induced liver injury, while serum IL-6 and TNF-α were more significantly reduced by EV treatment^[43]^. While EV show clear effects, it is unclear how their concentrations compare to the amount of *A. muciniphila *cells used in these studies, what the active EV components are, and how physiologically relevant these findings are for the *in vivo *situation.

## EXTRACELLULAR AND SECRETED PROTEINS

### PAS protein Amuc_1100

In search of proteins that interact with the host to exert beneficial effects, Ottman *et al.* identified the proteins encoded by a specific Type IV pili gene cluster (Amuc_1098 - Amuc_1099 - Amuc_1100 - Amuc_1101 - Amuc_1102) in fractions of *A. muciniphila *cultures that were enriched for outer membrane proteins^[48]^. Of note, the protein encoded by the Amuc_1098 locus was found to be the secretin PilQ located in the outer membrane. PilQ forms a ring-shaped pore required for the biosynthesis of Type IV Pili. It has been well established that Type IV Pili are abundant in the human intestinal microbes and are important for crosstalk with the host^[49]^. In addition, deep metaproteome analysis of fecal samples of a healthy subject with high levels of *Akkermansia *spp. showed the presence of the Amuc_1100-encoded protein. In contrast, Amuc_1100 was absent in fecal samples from an obese subject^[50]^. This 33 kDa protein was found to contain a canonical secretion signal sequence at the N-terminus and was defined as an outer membrane protein^[48]^. Recent results confirmed its location on the Type 4 Pili detected in *A. muciniphila* strain MucT [de Vos WM, personal communication]^[3]^. As discussed below, Amuc_1100 protein is involved in host interaction, and hence it is termed here PAS protein.

Many of the beneficial effects of both live and pasteurized *A. muciniphila *administration in HFD-fed mice could be replicated by administration of an N-terminal His-tagged purified PAS without signal sequence produced by and purified from *E. coli *(originally described as Amuc_1100* and hence termed here PAS*). HFD-fed mice treated daily with an oral gavage containing 3 μg of PAS* (the equivalent of 1.5 × 10^8^ CFU *A. muciniphila*) showed reduced body weight gain and plasma high-density lipoproteins and improved glucose tolerance. Treatment with PAS* restored plasma lipopolysaccharide (LPS) levels that were elevated in the HFD mouse model to those observed in mice fed normal chow, indicating an improvement of the gut barrier function. In addition, jejunal expression of tight junction proteins occludin and claudin 3 were increased in PAS*-treated mice at a higher level than in (pasteurized) *A. muciniphila*-treated mice^[14]^. The molecular basis of this barrier function increase was discovered to be through the activation of the Toll-like receptor 2 (TLR2) [Figure 2]. PAS*-induced TLR2 activation was much higher than activation by other *A. muciniphila* proteins that were produced in the same way by *E.coli*. As expected, PAS* activated TLR2 in a similar manner as *A. muciniphila *cells^[14,48]^. Structural analysis revealed that PAS* consists of a four-stranded antiparallel β-sheet and four α-helices. The four-stranded antiparallel β-sheet form two “αββ” motifs with 2 C-terminal helices. Interestingly, PAS* has been shown to bind directly to the purified TLR2 protein, further confirming its signaling function. Furthermore, PAS* forms oligomers that were reported to improve its TLR2 binding affinity^[51,52]^. PAS* also increased transepithelial electrical resistance in Caco-2 monolayers^[53]^. Moreover, PAS* has immune signaling capacity and induced cytokines IL-1β, IL-6, IL-8, IL-10 and TNF-α production in human peripheral blood mononuclear cells (PBMCs)^[48]^. PAS likely does not activate TLR5, TLR9 or the NOD2 receptors, but a recent study suggests that PAS might bind TLR4 based on molecular modeling^[54]^. However, further *in vitro *studies are needed to confirm whether PAS can bind TLR4 and whether this interaction leads to signal transduction.

An important finding was that the purified form of PAS* was thermostable and that the majority of the proteins remained folded at 70 °C^[14]^. This finding rationalized the activity of pasteurized *A. muciniphila* cells where PAS stays functional while other potentially negative components may become inactivated. Altogether, these results and those summarized below provide a strong basis for the conclusion that PAS is the molecular effector in *A. muciniphila*, which strengthens the intestinal barrier function and might have an immunomodulatory role in balancing the gut ecosystem.

#### PAS improves obesity and diabetes-related symptoms

In addition to the HFD mouse model (also known as a diet-induced obesity model), PAS* was also tested in rats on a HFD treated with streptozotocin, a type 2 diabetes model. Daily treatment with 3 μg PAS* reduced weight gain and improved barrier function. Additionally, treatment with PAS* led to a decrease in plasma TNF-α and increased mucin secretion, possibly through the increase in the number of goblet cells^[55,56]^.

An alternative mode of delivery of PAS was studied in a zebrafish model where the fish were fed a HFD^[57]^. PAS was expressed on the surface of fish-derived *Lactococcus lactis *ZHY1 (termed here PAS-ZHY) to treat fatty liver and intestinal barrier damage that are widespread in farmed fish^[57,58]^. PAS-ZHY, but not the control *L. lactis* bacteria without PAS, lowered hepatic fat accumulation in HFD-fed zebrafish. This effect was likely due to decreased expression of transcription factors that regulate fatty acid and triglyceride synthesis, such as *PPARγ* and *SREBP-1c*, as well as lipid absorption genes *CD36 *and *FABP6.* PAS-ZHY also alleviated hepatic HFD-induced inflammation, indicated by a decreased expression of *TNF-a *and *IL-6*. In the gut, expression of tight junction proteins *TJP1a, claudina, claudin7, claudin7b, claudin11a, claudin12*, and *claudin15a *was increased in PAS-ZHY-treated zebrafish, indicating that also when decorated on the cell surface of *L. lactis* cells, PAS could strengthen the gut barrier function. While the PAS-ZHY production levels were not quantified, it is likely that this resembles the activity of PAS when presented by *A. muciniphila* on its Type IV pili to the host.

#### PAS alleviates colitis and colitis-associated colorectal cancer

*A. muciniphila* is significantly reduced in patients with IBD^[9]^, notably those with ulcerative Colitis^[8]^, as well as in mice with colitis or colitis-associated colorectal cancer (CAC). Hence it was recently investigated whether PAS* could improve colitis, as well as CAC, to a similar extent as pasteurized *A. muciniphila*^[59]^*. *Mice were given dextran sulfate sodium (DSS) to induce colitis, followed by azoxymethane to establish CAC. Daily treatment with 3 μg of PAS* reduced infiltration of macrophages and CD8^+^ cytotoxic T lymphocytes (CTLs) in the colon. In the spleen, CD16/32^+^ macrophages and PD-1^+^ CTLs decreased by treatment with PAS*. PAS* treatment could prevent tumorgenesis by decreasing colonic DNA damage, cell apoptosis and abnormal cell proliferation^[59]^.

Similarly, it was studied whether PAS* had an effect on tryptophan metabolism in DSS-induced colitis, as gut microbiota-derived metabolites such as essential amino acid tryptophan (Trp) metabolites have been implicated in the pathogenesis of IBD^[60]^. Trp metabolism in the gut comprises three major pathways: the microbial pathway, kynurenine pathway and serotonin pathway^[61]^. Daily gavage with 3 μg PAS* and live or pasteurized *A. muciniphila *could not restore the reduction in Trp metabolites involved in the serotonin pathway in DSS-treated mice. All treatments significantly elevated the serum levels of kynurenine, but they failed to affect the kynurenine-Trp ratio. In the colon, expression of kynurenine pathway genes was unaffected, indicating that PAS* might inhibit the hepatic kynurenine pathway but not the colonic kynurenine pathway. However, all treatments restored the enrichment of Trp metabolism by the gut microbiota. Aryl hydrocarbon receptor targeted genes, including *Cyp1a1*, *Il-10 *and *Il-22*, were upregulated by all treatments. This indicates that colonic inflammation was decreased by the activation of the aryl hydrocarbon receptor by Trp metabolites, as has previously been described for Bifidobacteria and other bacterial species^[62-65]^.

Serotonin in the gut is mainly produced by the enterochromaffin cells (EC) in the intestinal mucus layer and is an essential signaling molecule that modulates gastrointestinal motility and immune responses^[66]^. Expression and activity of the serotonin reuptake transporter (SERT), which can remove serotonin from the interstitial place and terminate its signal, is inhibited by TLR2 activation^[67]^. Since the gut microbiota affects serotonin levels, Wang *et al. *investigated whether *A. muciniphila *could affect serotonin synthesis through the activation of TLR2 by PAS. PAS was produced in *E. coli* and purified using His-tag purification similar to previous studies, after which the His-tag was removed using cleavage by the tobacco etch virus (TEV) protease (termed here PAS^#^). In RIN-14B cells, a model for EC cells, 20 μg/mL PAS^#^ stimulated expression of *Nfkb1* and the serotonin rate-limiting enzyme *Thp1*, which could be inhibited by pre-treatment with a TLR2 inhibitor. In Caco-2 cells, PAS^#^ inhibited SERT expression through activation of TLR2. Similar results were found in the colon of antibiotic (Ab)-treated mice, which exhibit reduced serotonin concentrations in the gut, that were gavaged daily with 100 μg of PAS^#^*.* The increased colonic serotonin levels restored the lowered defecation rate and fecal water content in Ab-treated mice^[68]^. However, in colitis mice, PAS* failed to restore the reduction of serotonin*. *Serotonin synthesis genes were unaffected in the colon of PAS^*^-treated mice, which suggests that PAS^*^ does not regulate the serotonin pathway during colitis^[62]^.

#### PAS has antidepressant properties in animal anxiety and depression models

A recent study addressed the effect of PAS on serotonin and studied whether PAS had antidepressant activity in mice under chronic unpredictable mild stress (CUMS), a model for depression^[69]^. PAS was produced in *E. coli* and purified using His-tag purification similar to previous studies, after which the His-tag was removed using TEV (PAS^#^). It was found that treatment with 80 μg PAS^#^ had a tendency to improve depression-like behavior. Serotonin concentrations were increased in the gut, serum and the dorsal raphe nucleus of the central nervous system. In the hippocampus, PAS^#^ reversed the down-regulation of brain-derived neurotrophic factor and inflammatory cytokines induced by CUMS, which might partially explain its antidepressant effect^[69]^. Similar results were found in a study where a broad-spectrum cocktail mixture was used to increase anxiety and depression-like behavior in mice^[70]^.

Interestingly, a form of PAS^#^, in which the 80 N-terminal residues were removed, induced a slightly higher upregulation of *Tph1 *in RIN-14B cells compared to full-length PAS^#^. This PAS^Δ80^ was found to readily dimerize and had an increased affinity for TLR2^[51]^. This is in line with the observation that the TLR2 receptor is a dimer with either TLR1 or TLR6, but presently it is not known which dimer is the *in vivo *PAS receptor.

In CUMS, daily gavage with 80 μg PAS^Δ80^ reduced anxiety and depressive behavior to a larger extent compared to 80 μg PAS^#^. Serum levels of serotonin were restored after treatment with PAS^#^ or PAS^Δ80^, and mRNA levels of IL-1β, IL-6, and TNF-α were significantly lower in the ileum, colon, serum and frontal cortex. Both treatments counteracted hypothalamic-pituitary-adrenal axis hyperactivity, reducing the levels of glucocorticoid receptor in the hippocampus and of serum corticosterone^[71]^.

#### PAS attenuates mucositis

Besides colitis, PAS was shown to have beneficial effects on mucositis. Mucositis occurs in the mucosa of the gastrointestinal tract when oncology patients receive chemotherapy such as 5-Fluorouracil (5-FU), which triggers inflammatory responses and induces intestinal mucosal damage. Chen *et al. *found that daily prophylactic treatment with 100 μg PAS* or *A. muciniphila* could attenuate the upregulation of IL-6, TNF-α and NLRP3 inflammatory vesicle activation in 5-FU-treated mice^[72]^. Both treatments prevented shortening of the colon and lowered the diarrheal toxicity score and colonic histological pathology score, indicating amelioration of mucositis. Expression levels of tight junction proteins *ZO-1*, O*ccludin*, and *Claudin-1 *were restored, which may explain the amelioration of inflammation.

#### PAS reduces periodontitis

In the oral cavity, PAS* showed anti-inflammatory effects in *Porphyromonas gingivalis*-induced experimental periodontitis. This chronic inflammatory disease is characterized by an increase in M1 macrophages in the oral cavity, which disrupts the M1/M2 macrophage ratio, creating a hyperinflammatory environment^[73]^. *A. muciniphila *may be present in the oral cavities of healthy individuals but is absent in patients suffering from severe periodontitis^[74]^.

Daily intraoral gavage of 6 μg PAS* reduced periodontitis-induced alveolar bone loss and lowered the M1/M2 macrophage ratio in the submandibular lymph nodes in *P. gingivalis*-treated mice^[75,76]^. This lowered ratio is crucial for wound healing^[77]^. Additionally, PAS* increased the expression of IL-10 and of the T-cell chemoattractant CXCL10 in gingival tissues. This was confirmed *in vitro* where PAS* increased IL-10 secretion and decreased TNF-α secretion in bone marrow-derived macrophages infected with *P. gingivalis*^[76]^. This study shows that the effects of *A. muciniphila *are not limited to its direct effect on *P. gingivalis*^[75]^, but that it also modulates the oral immune response^[72]^.

#### PAS can strengthen IL-2 based immunotherapy antitumor effects

The cytokine IL-2 is used as immunotherapy to treat various cancers. However, there is a large interindividual variability in the response to this treatment, which might be attributed to interindividual differences in gut microbiota. Patients that responded to ICI treatment were found to have a higher abundance of *A. muciniphila *and treatment with *A. muciniphila *in different mouse models could restore ICI response^[27-30]^. Similarly, *A. muciniphila *strengthened IL-2 antitumor effects in subcutaneous melanoma and colorectal tumor-bearing mice^[32]^. Interestingly, this effect could be replicated by daily treatment with 10 μg PAS*. *In vitro *experiments showed that the antitumor effects of PAS* were partly mediated through TLR2 activation^[32]^.

#### PAS as the main effector of A. muciniphila related therapeutic effects?

PAS can replicate a great number of the beneficial effects of *A. muciniphila* on gut permeability and health. However, it remains unclear whether other factors might play a role. The PAS* protein used in most of these studies still contains the N-terminal His-tag label attached and is the same as described in the original discovery^[14,48]^. This His-tag label might interfere with folding or activity of the protein, although when removed as in PAS^#^, its signaling activity is maintained. Several studies administered 3 μg of PAS* daily to the animal models, estimated to be equivalent to 1.5 × 10^8^ CFU of *A. muciniphila. *These concentrations are likely representative of physiological concentrations in the gut, as production levels of PAS by *A. muciniphila* are high^[3,14]^. Other studies use much higher concentrations, up to 100 μg PAS* per day, although it is not clear whether identical quantification methods have been applied. While the findings of these studies still show the therapeutic relevance of PAS, it is unclear whether the same effects would be observed with more physiologically relevant concentrations. It would be interesting to measure PAS concentrations in the gut and the mucus layer in health and disease and to study the potential dose-dependent effects of PAS in different diseases. Ideally, future studies using His-tag purification to obtain PAS protein should remove the His-label from the protein before studying its effects.

### Glucagon-like peptide-1-inducing protein P9 Amuc_1631

While the recent findings of PAS are promising and replicate the activity of live and pasteurized *A. muciniphila *cells, another interesting protein was recently identified that could play a role in the prevention of obesity by *A. muciniphila.*

By replicating the early experiments that defined the role of *A. muciniphila* in improving barrier function, it was found that oral administration of *A. muciniphila *increased β-oxidation genes in HFD-fed mice^[10,78]^. The mass and size of the interscapular brown adipose tissue (iBAT), which mediates non-shivering thermogenesis, were decreased, while the mass and size of the epididymal white adipose tissue was unaffected. Additionally, plasma GLP-1 levels, which can regulate BAT thermogenesis^[79]^, as well as ileal expression of *Gcg* (which encodes glucagon or GLP-1), were found to be increased. By creating a series of* A. muciniphila* supernatant fractions based on size^[48]^, testing their effect on GLP-1 secretion of L-cells and subsequent liquid chromatography coupled with tandem mass spectrometry, a selection of nine potential GLP-1 inducing proteins were identified. Screening of this selection identified the P9 protein (encoded by the gene with the Amuc_1631 locus and overproduced as a C-terminal His-tag purified protein from* E.coli*, denoted here as P9*), as one of the most potent GLP-1 secretion inducers *in vitro*. Mice that were injected intraperitoneally with 100 μg P9* showed weight loss and a decrease in glucose intolerance. In HFD-fed mice, oral administration of 100 μg P9* reduced weight gain, food intake, adipose tissue volume and glucose intolerance and stimulated *Gcg *expression in the ileum. P9* induced thermogenesis, indicating that P9* prevents obesity by regulating glucose homeostasis and inducing thermogenesis by iBAT.

*In vitro*, P9* increased the expression of phosphorylated cAMP-response-element-binding protein and phosphorylated heat shock protein 27 and increased calcium influx in L cells. Using ligand-receptor capture technology, ICAM-2 was identified as the receptor that binds P9*, leading to its observed effect [Figure 2]. Additionally, both GLP-1 receptor signaling and IL-6, which can also stimulate GLP-1 secretion^[80]^, were also involved in iBAT activation and thermogenesis. The circulating GLP-1 concentration was substantially increased by the P9* treatment in wild-type mice but not in IL-6-KO mice^[81,82]^.

Based on proteome analysis of mucus-grown *A. muciniphila* cells, the amount of the P9 protein is approximately three-fold lower compared to that of PAS, whereas its size is three-fold larger, suggesting that there may be close to 10-fold more PAS than P9 molecules per cell [Table 2]. Hence, it appears that this study also used supraphysiological concentrations of the protein of interest in their animal studies^[81,84]^. Therefore, the physiological relevance of their findings remains unclear. The pharmacological use of P9 in improving obesity and its related metabolic symptoms is shown, but it will be interesting to study the relevance of P9 in the health effects of *A. muciniphila*. A first step would be to investigate whether P9 is also thermostable and might contribute to the observed effects of pasteurized *A. muciniphila*. Moreover, the localization of P9 needs to be addressed to determine whether it is completely secreted, linked to the outer membrane or in another form accessible to the host cells. In a previous study, P9 was only found in fractions containing cytoplasmic and membrane proteins of *A. muciniphila*, and not in outer membrane fractions^[48]^. Moreover, next to P9, various other proteins were enriched in the purified *A. muciniphila* protein fraction, which might also contribute to the potential mode of action of *A. muciniphila*. Finally, in the reported mouse and human studies with pasteurized or live *A. muciniphila*, no indications of GLP-1 induction have been reported, which questions the relevance of P9 as a signaling protein^[41,83]^.

**Table 2:** Production and predicted location of the different signaling proteins of *A. muciniphila*. Data are based on global proteome data of *A. muciniphila* cultures growing mid-exponentially on mucin^[38]^. Indicated molecular sizes of the proteins are based on the predicted amino acid sequences. The signal peptide presence was predicted by SignalP 4.0^[83]^

**Name/function**	**Gene**	**Accession number**	**Size (kDa)**	**Protein production level** **(logCPM)^[38]^**	**Signal peptide present**
PAS	*Amuc_1100*	B2UR41	34.2	10.35	Yes
P9	*Amuc_1631*	B2UM07	83.9	9.87	Yes
Predicted protein	*Amuc_1409*	B2UKV9	16.5	7.93	Yes
β-N-acetyl-hexosaminidase	*Amuc_2109*	B2UPP0	38.4	N.D.	No
Aspartic protease	*Amuc_1434*	B2UKY3	35.9	2.46	Yes
Acetyltransferase	*Amuc_2172*	B2UPV1	19.3	N.D.	No

kDa: Kilodalton; LogCPM: logarithm of counts per million reads; N.D.: not detected.

### Other signaling proteins without reported mode of action

Another secreted protein of *A. muciniphila* was recently described that might aid in the treatment of colitis. A protein encoded by the locus tag Amuc_1409 was purified with an N-terminal His-tag from overproducing *E.coli* and was found to stimulate growth and increase the number of lobes in murine as well as human intestinal organoids. When human intestinal organoids were treated with proinflammatory cytokines to simulate IBD *in vitro*, treatment with Amuc_1409* protein increased the surface area compared to vehicle-treated organoids. Expression of proliferation marker Ki-67, intestinal stem cell marker ASCL2 and tight junction protein ZO-1 were all significantly increased. In mice with DSS-induced colitis, treatment with 3.6 μM Amuc_1409* per day induced body weight gain, increased colon length and lowered the histopathological score in the distal colon. Additionally, expression of stem cell-related genes in the distal colon was higher in Amuc_1409*-treated mice compared to vehicle-treated mice^[85]^.

Much remains to be investigated about the Amuc_1409 protein, but the first results seem promising. However, the abundance of the Amuc_1409 protein is 263 times lower than PAS [Table 2], so it is unclear whether the concentrations used in this study are representative of the physiological concentrations in the gut. It remains to be determined whether Amuc_1409 protein is thermostable and whether it is only present as a secreted protein, as is reported.

The protein encoded by locus tag Amuc_2109 is a β-N-acetylhexosaminidase and might release β-linked N-acetylglucosamine (GlcNAc) or N-acetylgalactosamine (GalNAc) terminals with important biological functions from the mucus layer. Qian *et al. *hypothesized that the Amuc_2109 protein purified with a C-terminal His-tag from overproducing *E.coli* might have beneficial effects on gut health, as the mucus layer is important in maintaining the integrity of the intestinal barrier*. *Therefore, DSS-treated mice were gavaged with 20 μg Amuc_2109* protein or Amuc_2109^ΔD246A^ protein (derived from a mutated Amuc_2109, predicted to be lacking its catalytic amino acid). Body weight loss, disease activity index and colonic histopathological damage decreased while colon length increased in mice treated with Amuc_2109* protein, indicating that it could alleviate colitis-associated symptoms. Expression levels of tight junction proteins ZO-1, occludin, and claudin-1, as well as of the proinflammatory cytokines IL-6, TNF-α and IL-1β, were restored and activation of NLRP3 inflammatory vesicles was reduced. The inactive Amuc_2109^ΔD246A ^had no such effects^[86]^.

Of note, Amuc_2109 was not detected in *A. muciniphila *grown on mucin [Table 2]. Additionally, Amuc_2109 has no canonical signal sequence, so it is unlikely to be secreted when it is produced. It is thus unlikely that Amuc_2109 would reach high concentrations in the gut, and it likely does not play a major role in the observed health benefits of *A. muciniphila.*

The protein encoded by the gene with locus tag Amuc_1434 is an aspartic protease that can degrade MUC2, the main component of the colorectal mucus layer. MUC2 plays a role in the development of colorectal cancer, which led Meng *et al. *to study the effect of Amuc_1434 protein, also purified as a His-tag protein from overproducing *E.coli*, on colorectal cancer LS174T cells. Proliferation of LS174T was significantly inhibited starting from Amuc_1434* protein concentrations of 8 μg/mL. Amuc_1434* protein induced G0/G1-phase cell-cycle arrest in LS174T cells and increased the expression of the tumor suppressor gene p53, which controls the initiation of the cell cycle and promotes apoptosis. Apoptosis was likely promoted through upregulation of tumor necrosis factor-related apoptosis-inducing ligand (TRAIL) and its receptors DR4 and DR5, and caspase 3 and 8^[87]^. In conclusion, Amuc_1434* protein suppressed LS174T cell viability via TRAIL-mediated apoptosis pathway.

Amuc_1434 protein concentrations were 10 million times lower compared to PAS in *A. muciniphila *grown on mucin [Table 2]. Of note, Amuc_1434 has a predicted signal sequence, so it is possibly secreted in the gut. However, it remains to be determined whether its expression and secretion might be upregulated under specific conditions to a sufficiently high enough concentration to affect cancer cell proliferation *in vivo.*

Finally, the protein encoded by locus tag Amuc_2172 was recently shown to reprogram the tumor microenvironment in a mouse model of colorectal cancer^[88]^. Since EV of *A. muciniphila *prevented colitis-associated tumorgenesis in DSS-treated mice, the authors aimed to identify a bioactive component in the EV that could be responsible for this effect. *In vitro* studies identified the protein fraction with a size below 30 kDa as bioactive, and mass spectrometry analysis showed that the acetyltransferase encoded by Amuc_2172 was present in the EV. Amuc_2172* could inhibit cancer cell growth *in vitro* and daily peritumoral injection (150 μg/kg, 12 days) inhibited allografted tumor growth *in vivo*. Intraperitoneal administration of Amuc_2172* had no effect on allografted tumors, while it could inhibit intestinal tumorgenesis in *Apc*^min/+ ^mice by promoting the function of CTLs. Amuc_2172* was found to promote HSP70 transcription and secretion via histone H3 acetylation both *in vitro *and *in vivo*. HSP70 knockdown and CTL depletion blocked the antitumor effect of Amuc_2172*, suggesting that Amuc_2172* inhibits tumorgenesis by inducing HSP70 secretion and CTL immune activity. Nevertheless, in a previous study, Amuc_2172 was not detected in *A. muciniphila *grown on mucin and it does not contain a canonical secretion signal sequence [Table 2]. Therefore, it remains to be conclusively verified whether or not Amuc_2172 contributes to potential antitumor effects of *A. muciniphila*.

## METABOLITES

### Short-chain fatty acids

Short-chain fatty acids (SCFAs) are well-studied microbial products that signal to the host. By activating their receptors FFAR2, FFAR3, HCAR2 and OLFR78 present in the gut, they can affect several gut functions such as gut hormone release, inflammation and motility^[89,90] ^[Figure 2]. *A. muciniphila* produces mainly acetate and propionate from mucus and thus might contribute to SCFA-mediated effects seen in the gut, aided by its localization close to the epithelial barrier and its upregulation in fasting conditions^[37,91]^.

However, since most health effects of *A. muciniphila *are still present with washed cells and after pasteurization, it is unlikely that SCFA signaling plays an important role.

### Harmaline

*A. muciniphila* may modulate host systemic antiviral immune responses through secretion of the metabolite harmaline, a β-carboline alkaloid, which activates the bile acid-TGF5-NF-κB signaling axis^[92] ^[Figure 2]. Patients that survived a rare condition known as Severe Fever with Thrombocytopenia Syndrome Virus (SFTSV) were found to have an increased fecal abundance of *A. muciniphila *compared to patients that succumbed to the disease. This increased abundance was inversely correlated with levels of proinflammatory cytokines IL-1β and IL-6 in serum. Fecal transplantation of recovered patients to an SFTSV mouse model increased the survival rate by 31%, while fecal transplantation of deceased patients had no effect on lethality. Similarly, treatment with live *A. muciniphila *increased survival rate, but surprisingly, pasteurized *A. muciniphila *had no effect.

By comparing the results of untargeted metabolomics analyses of serum samples of surviving patients, deceased patients and healthy controls, the bile acids (BAs) chenodeoxycholic acid (CDCA), GCDCA, TCDCA and taurodeoxycholic acid (TDCA) were found to be upregulated in the surviving patients. Furthermore, treatment of PBMC in patients with these bile acids led to a decrease in IL-6.

Based on these findings, it was hypothesized that a secreted metabolite that upregulates BAs might be responsible for the observed effects. Therefore, supernatant of *A. muciniphila* cultures was divided into fractions based on molecular weights^[81]^. Mass spectrometry analysis identified five metabolites that were present at high concentrations in the fraction that showed the highest induction of bile acid-CoA:amino acid N-acyltransferase (BAAT) expression in the hepatic Huh-7 cell line. From these five metabolites, harmaline showed the highest induction of BAAT in both Huh-7 cell line and mouse primary hepatocytes. Germ-free mice and the SFTSV model mice colonized with *A. muciniphila* had picomolar quantities of harmaline in serum. Furthermore, oral treatment with harmaline increased the survival rate in the SFTSV mouse model^[92,93]^.

It is not clear how much harmaline is produced by *A. muciniphila* and which genes are involved in the production of harmaline in *A. muciniphila. *Moreover, it remains to be determined how bile acids regulate the suppression of NF-κB during SFTSV. Nevertheless, harmaline shows promise as a pharmaceutical against SFTSV, although it is a controlled substance. Previous results already showed that harmaline could have an antiviral effect against Herpes Simplex Virus^[94-96]^. This study highlights that the effects of *A. muciniphila *in specific diseases might be mediated by different factors, as pasteurized *A. muciniphila *had no effects. However, further research is warranted to confirm that *A. muciniphila *produces significant amounts of harmaline, how unique this is as harmaline is produced in many biological systems, and whether it can be used as a therapeutic in SFTSV.

### ADH-heptose-like soluble metabolite

A recent *in vitro *study showed that *A. muciniphila *can also activate NF-κB independent of MYD88-dependent TLRs or Nucleotide Oligomerization Domain proteins (NOD). A soluble metabolite of *A. muciniphila* was found to intracellularly activate the newly described pattern recognition receptor alpha kinase 1 (ALPK1) in intestinal epithelial cells, leading to improved intestinal barrier function^[97]^. Since ALPK1 senses ADP-heptose, an intermediate of the LPS biosynthetic pathway, the authors investigated whether *A. muciniphila* could produce ADP-heptose and whether this was the soluble metabolite that activated ALPK1. They found that the enzyme responsible for ADP-heptose production, HldE (Amuc_0415), is present in the genome of *A. muciniphila*. Cell lysates of *E. coli *heterologously expressing this HldE could activate NF-κB in a ALPK1-dependent manner. In addition, similar to ADP-heptose, the *A. muciniphila*-derived NF-κB-activating molecule was partially inactivated by phosphodiesterase from *Crotalus adamanteus*.

## CELL ENVELOPE FRAGMENTS

Cell envelope fragments of *A. muciniphila*, such as peptidoglycans or membrane lipids, can be released in the gut due to normal processes such as cell division, growth or death. Cell envelope fragments of Gram-negative bacteria can trigger anti-bacterial responses and inflammation, yet recent studies indicate that cell envelope fragments from *A. muciniphila *could also have anti-inflammatory effects^[98,99]^.

### Peptidoglycan muropeptides

Peptidoglycan (PG) is a cell envelope component that consists of 1,4-linked β-D-N-acetylglucosamine (GlcNAc) and β-D-N-acetylmuramic acid (MurNAc) linked by short peptides. During growth and cell division, Gram-negative bacteria release fragments of PG, which can function as signal molecules to the host as microbe-associated molecular patterns (MAMPs). MAMPs are recognized by NOD1 and NOD2 and other immunologic receptors, activating an antimicrobial response^[100]^. A recent study reports the isolation of PG fragments (i.e., muropeptides) from *A. muciniphila *and analyzed their structure and immunologic signaling potential. Structurally, the PG of *A. muciniphila *contained non-acetylated glucosamine, which seems a novel component of the PG of Gram-negative bacteria. The *A. muciniphila *muropeptides stimulated NOD1 and NOD2, activating NF-κB to a similar extent as *E. coli *muropeptides^[98] ^[Figure 2]. Interestingly, these findings contradict the potential role of PG non-*N*-acetylation as an evasion mechanism from NOD1, shown in *L. monocytogenes*^[101]^. More research is needed to further elucidate the effect of PG on gut health. Of interest, muropeptides were recently shown to modulate the response to anti-PD1 checkpoint inhibitors in cancer immunotherapy^[102]^. Therefore, it was hypothesized that *A. muciniphila* muropeptides also contribute to the efficacy-promoting effect of *A. muciniphila *in anti-PD-1 cancer immunotherapy, but considerable work has to be done to validate this^[30]^.

### Membrane lipids

Recently, a cell membrane diacyl phosphatidylethanolamine (12-methyltetradecanoyl-13-methyltetradecanoyl-sn-glycero-3-phosphoethanolamine, diacyl PE) was identified in the cell membrane of *A. muciniphila *using lipid extraction, chromatographic separations and screening for induction of cytokine release from murine bone marrow-derived dendritic cells*. *The most active fraction was analyzed by mass spectroscopy and nuclear magnetic resonance, identifying the diacyl PE as the major component. This diacyl PE was different from other diacyl PEs forming the cell membrane of most microbial members of the gut microbiota. Knockdowns of TLR6 and TLR1 showed that a TLR2-TLR1 heterodimer was required for TNF-α induction by 50 μg/mL of diacyl PE in human monocyte-derived dendritic cells^[99] ^[Figure 2]. It will be interesting to compare the effects of this phospholipid with the effect of *A. muciniphila *in *in vivo *models, at concentrations that are in accordance with the amount of the phospholipid present in the membrane of the cells.

Using quantitative trait locus mapping of the genomes of a genetically diverse mouse population, combined with fecal metagenomics and lipidomics, a recent study reported the identification of ornithine lipid (OL) as another potential immunomodulatory effector molecule produced by *A. muciniphila*^[103]^. OLs are found in the outer membrane of Gram-negative bacteria^[104]^ and OLs were identified as a dominant lipid species in *A. muciniphila* cell extracts. Caecal *A. muciniphila *levels correlated with the abundance of OLs. Furthermore, when inoculating germ-free mice with different Gram-negative species, only inoculation with *A. muciniphila* led to detectable OLs levels in the caecum, indicating that *A. muciniphila* likely is an important producer of OLs in the gut. *In vitro*, OLs could prevent LPS-induced inflammation and induced IL-10 in bone marrow-derived macrophages. In the gut, OLs produced by *A. muciniphila *might similarly regulate LPS-induced inflammation by upregulating Gene Activating transcription factor 3 (*Atf3*), a transcription factor that negatively regulates proinflammatory cytokine transcription, when the latter is induced by TLR4 activation^[103]^.

## FUTURE PROSPECTS

Thanks to the large amount of attention that *A. muciniphila *continues to receive, several promising proteins and other molecules have been identified that can prevent or potentially treat metabolic disorders and other diseases. While it is tempting to use high and supraphysiological concentrations when testing a newly identified compound to ensure that an effect will be observed, from a microbiological standpoint, it would be more interesting to use concentrations that are physiologically representative of the *in vivo *situation in the gastrointestinal tract in order to unravel the mode of action of *A. muciniphila*. Therefore, it remains important to carefully consider which concentrations to administer in mouse models, and to consider extensive dose-response studies. Furthermore, since pasteurized *A. muciniphila *retains its beneficial effects in mice and humans, it would be relevant to consider testing new components for their thermostability and to investigate whether a compound is present in or on the cell, or if it is secreted.

Since a method to efficiently alter the genome of *A. muciniphila *is still lacking, comparing the effects of live and pasteurized cells, extracellular vesicles and lysates might give more insight into which cellular compounds are important in *A. muciniphila*’s effects as a next-generation beneficial microbe. ^[105]^ Furthermore, elucidating the complex function of the gut barrier might provide further insights into whether *A. muciniphila *exerts its beneficial effects by counteracting gut permeability and what other mechanisms have developed via its symbiotic association during evolution.

## DECLARATIONS

### Copyright

© The Author(s) 2023.
